# Complex roles of TGF-β signaling pathways in lung development and bronchopulmonary dysplasia

**DOI:** 10.1152/ajplung.00106.2021

**Published:** 2023-01-10

**Authors:** Rebecca J. Calthorpe, Caroline Poulter, Alan R. Smyth, Don Sharkey, Jayesh Bhatt, Gisli Jenkins, Amanda L. Tatler

**Affiliations:** ^1^Lifespan & Population Health, School of Medicine, University of Nottingham, Nottingham, United Kingdom; ^2^NIHR Nottingham Biomedical Research Centre, Biodiscovery Institute, University of Nottingham, Nottingham, United Kingdom; ^3^Department of Pediatrics, Queens Medical Centre, Nottingham University Hospitals NHS Trust, Nottingham, United Kingdom; ^4^Centre for Perinatal Research, School of Medicine, University of Nottingham, Nottingham, United Kingdom; ^5^National Heart and Lung Institute, Imperial College London, London, United Kingdom

**Keywords:** bronchopulmonary dysplasia, transforming growth factor-β

## Abstract

As survival of extremely preterm infants continues to improve, there is also an associated increase in bronchopulmonary dysplasia (BPD), one of the most significant complications of preterm birth. BPD development is multifactorial resulting from exposure to multiple antenatal and postnatal stressors. BPD has both short-term health implications and long-term sequelae including increased respiratory, cardiovascular, and neurological morbidity. Transforming growth factor β (TGF-β) is an important signaling pathway in lung development, organ injury, and fibrosis and is implicated in the development of BPD. This review provides a detailed account on the role of TGF-β in antenatal and postnatal lung development, the effect of known risk factors for BPD on the TGF-β signaling pathway, and how medications currently in use or under development, for the prevention or treatment of BPD, affect TGF-β signaling.

## Graphical abstract

**Figure:**
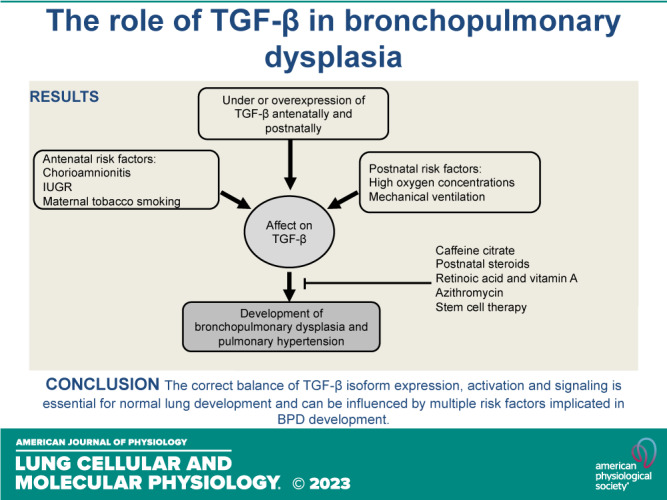

## INTRODUCTION

Bronchopulmonary dysplasia (BPD) was first described by Northway and colleagues in 1967 as a severe form of chronic lung disease affecting mostly preterm infants (1, 2). Postmortem lung samples of these infants showed hypertensive pulmonary vascular remodeling, large airway smooth muscle (ASM) hyperplasia, and heterogeneity of the parenchyma with diffuse fibroproliferative changes (3, 4). Commonly, such pathological changes are referred to as “old” or “classical” BPD. Recent advances in neonatal care have led to significantly improved survival for preterm infants, most markedly for those at <26 wk gestation (5). With this, a “new” form of BPD has emerged, primarily related to extreme prematurity, due to the disturbance of lung development during the critical period of saccular lung development (1, 3). Fibrosis is a less prominent feature and “new” BPD is instead characterized by more homogenous lung parenchyma with a larger, simpler alveolar structure and mild airway muscle thickening (1, 3).

The transforming growth factor-β (TGF-β) superfamily of growth factors are widely expressed proteins with well-known and diverse roles in development, wound healing, and fibrosis. TGF-β superfamily members have been implicated in various stages of lung development in utero and postnatally and in the pathogenesis of many of the features of both “new” and “old” BPD including parenchymal fibrogenesis, remodeling of the pulmonary vasculature and ASM remodeling. In this review, we aim to provide a comprehensive overview of the various roles of TGF-β proteins in normal lung development and BPD pathogenesis, with a particular focus on the isoforms of TGFβ1–3. By reviewing recently published research, we will explore the relationship between some known risk factors that contribute to the development of BPD with TGF-β proteins and the pathological features of the disease.

## CONSEQUENCES OF BPD

**Figure 1.**
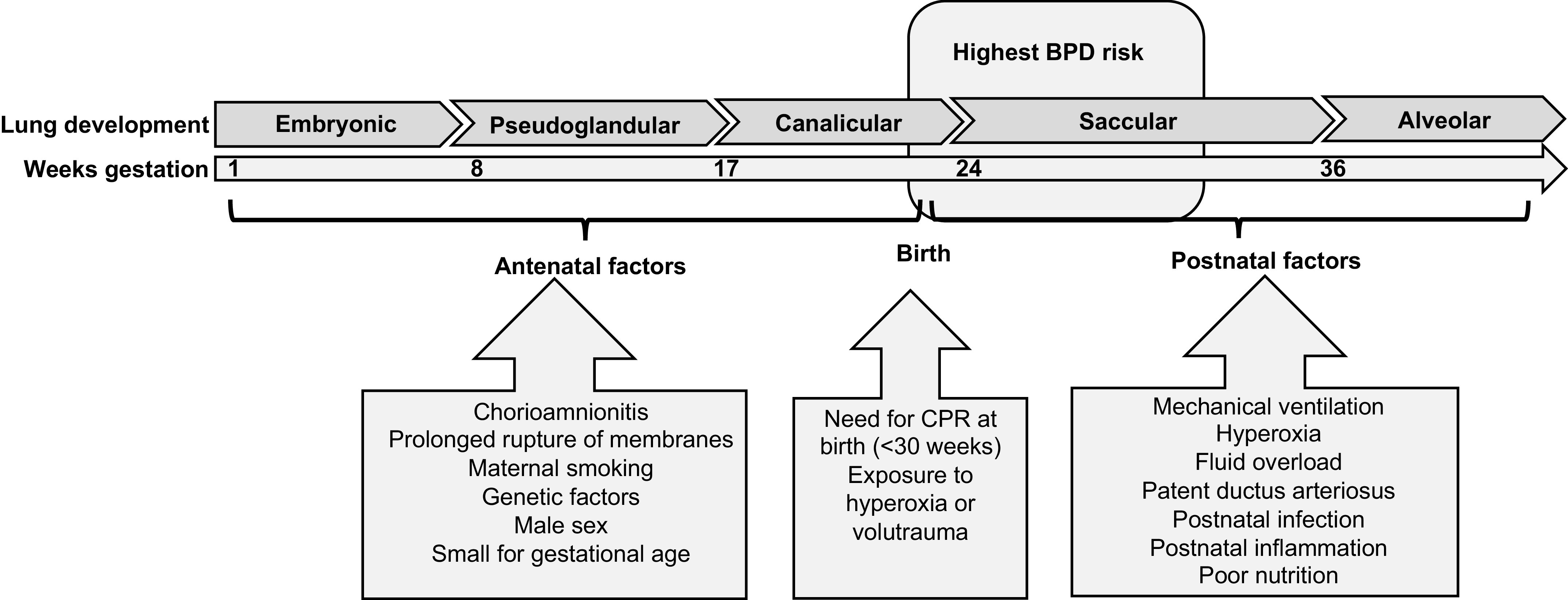
Risk factors associated with the development of bronchopulmonary dysplasia. [Adapted from Davidson and Berkelhamer (7) under an open access Creative Common CC BY license].

Despite survival for extremely preterm infants improving, rates of BPD among these infants have also increased, with an overall increase of 4.2% in a review of 11 high-income countries (6). There are numerous risk factors for BPD development, which are highlighted in Fig. 1 (7–10). Antenatal factors include male sex, being small for gestational age, genetics, maternal smoking, and chorioamnionitis. At birth and postnatally, BPD risk is associated with extreme preterm birth, the need for cardiopulmonary resuscitation (<30 wk), mechanical ventilation, exposure to hyperoxia, or volutrauma as a result of mechanical ventilation, as well as postnatal infection and/or inflammation (8–11).

BPD can have significant health implications not just in the neonatal period but throughout childhood and adulthood. Long-term sequalae include adverse respiratory, cardiovascular, and neurological outcomes. Infants with BPD have an increased risk of substantial airway impairment with airway obstruction on pulmonary function testing, a higher risk of airway hyper-responsiveness and asthma-like symptoms, and reduced respiratory reserve persisting into adolescence and adult life (1, 12–15).

Pulmonary hypertension (PH) affects 8%–25% of infants with BPD and is characterized by abnormal vascular remodeling and vascular growth arrest resulting in increased pulmonary vascular resistance (16). Crucially, it has been shown that early disruption of vascular growth contributes to reduced alveolarization, which is a feature of BPD (17), in addition to leading to the development of PH. The incidence of PH-associated BPD rises with increasing BPD severity (18). This is of particular clinical importance given the associated increased mortality, need for tracheostomy, worse neurodevelopmental outcomes, and feeding problems in these infants (13, 14, 16, 19). Improved understanding of the mechanisms driving normal lung growth and the development of BPD are therefore essential.

## NORMAL LUNG DEVELOPMENT

Lung development is typically divided into 5 stages consisting of embryonic (4–7 wk), pseudoglandular (5–17 wk), canalicular (16–26 wk), saccular (24 wk to birth), and alveolar (from 36 wk) (20) (Fig. 1). During branching morphogenesis, the lung bud undergoes a dichotomous pattern of division of the airways forming terminal bronchioles during the pseudoglandular stage, which further divide in the canalicular stage leading to the formation of respiratory bronchioles. The saccular stage is characterized by the development of the primitive lung saccules, lined by type 1 and 2 alveolar cells, thinning of the connective tissue between the airspaces and capillaries, and initiation of surfactant production (1, 13, 21). Alveolar development is the final stage of lung development occurring from 36 wk gestation until early childhood and is characterized by secondary septation of the primitive lung saccules leading to alveolarization.

This branching morphogenesis acts as a template for pulmonary vasculature growth, which follows a similar branching process during embryological development. Vasculogenesis predominantly occurs up to 17 wk gestation with the formation of extrapulmonary, lobar, and pre-acinar arteries. From the canalicular phase, angiogenesis occurs with the formation of intra-acinar arteries (18–25 wk), alveolar arteries (25 wk onward) and capillary alveoli (30 wk onward) (22).

The complex nature and relatively late timing of branching morphogenesis in both alveolar and vascular development are critical for infants who are born extremely premature. Crucially, alveolarization and angiogenesis are closely linked in lung development with inhibition of angiogenesis able to interrupt alveolarization (23). Furthermore, the lungs of infants born extremely premature are exposed to a complex interaction of perinatal and postnatal stressors during their subsequent neonatal care, which may disrupt normal alveolar and pulmonary vascular development and promote BPD pathogenesis (Fig. 1) (7).

## TRANSFORMING GROWTH FACTOR β SIGNALING IN LUNG DEVELOPMENT AND BPD

TGF-β exists as three isoforms, TGF-β1, TGF-β2, and TGF-β3, which are encoded by distinct genes. They belong to the TGF-β superfamily of proteins, which contains over 30 members including activins, bone morphogenetic proteins (BMPs), and growth and differentiation factors. TGF-β superfamily members have diverse functions in development, homeostasis, repair and disease, which signal through canonical (Smad signaling) and noncanonical signaling pathways (24–26). The Smad signaling pathway includes two distinct pathways *1*) the TGF-β-Smad pathway, which is mediated via Smad 2 and Smad 3 phosphorylation, and *2*) BMP-Smad pathway which involves Smad 1/5/8 phosphorylation (27–29). Both signaling pathways are critical for normal alveolar and pulmonary vasculature development (30–33) and have been implicated in the pathogenesis of BPD (34, 35).

Animal studies have given insights into the roles of TGF-β isoforms in lung morphogenesis. During normal lung development, TGF-β isoforms show different temporal expression patterns; TGF-β1 and TGF-β3 are expressed in early saccular development whereas TGF-β2 is expressed later in more mature epithelium (28). Furthermore, TGF-β isoform-specific null mice have helped shed light on the functional consequences of TGF-β isoforms on lung development (Table 1) (25, 34). TGF-β1 null mice have no overall lung developmental defects at birth (35) whereas TGF-β2 null mice have high perinatal mortality associated with dilated conducting airways and collapsed distal airways collapsed (36), and TGF-β3 null mice die within hours of birth exhibiting severely delayed lung development (37).

**Table 1.** Expression of TGF-β isoforms and associated KO phenotypes in mice

Isoform	mRNA Location	Location within the Lung	KO Mice Phenotype
TGF-β	Endothelial, hematopoietic, neural cells, connective tissue	Throughout the mesenchyme, highly localized at the epithelial branching points	Systemic inflammation, perivasculitis and lymphocytic infiltration in the lungs. High mortality at weaning.
TGF-β2	Epithelial and neural cells	Localized in the distal epithelium	Cardiac, spinal column, urogenital, eye, and ear abnormalities. Dilation of the conducting airways and collapsed distal airways. High mortality prior and soon after birth.
TGF-β3	Mesenchymal cells	Localized in the distal epithelium	Cleft palate development. Dilation of the conducting airways, alveolar hypoplasia and mesenchymal thickening. High mortality shortly after birth.

Sources: Refs. (25) and (34). KO, knockout; TGF, transforming growth factor.

Other studies have suggested that correct temporal antenatal TGF-β isoform expression is critical for lung development. Conditional mesenchyme-specific deletion of *TGF-β1* in the lung during early branching morphogenesis [*embryo day 7.5* (*E7.5*)] caused bilateral pulmonary hypoplasia with the pups dying within a few hours of birth, whereas deletion at the end of branching morphogenesis (*E15.5*) resulted in lungs that were of similar size and gross appearance to wild type lungs (38). Conversely, in primates, adenoviral-induced TGF-β1 overexpression during the later canalicular or saccular stages resulted in lung parenchymal hypoplasia and fibrosis of the interstitial reticulum, pleural membranes, and alveolar septa (39). Together, these studies indicate that correct early expression of TGF-β1 may be needed for normal lung development. It has been suggested that the lack of aberrant lung development in the TGF-β1 null mouse despite clear developmental effects in other models could be due to maternal transfer of TGF-β1 (40). In contrast, ex vivo tissue models have demonstrated that inhibition of TGF-β2 with antisense oligonucleotides can inhibit both early lung branching and secondary branching whereas inhibition of either TGF-β1 or TGF-β3 had no effect (41). Although it is clear that further research is needed to fully delineate the exact differential roles of the TGF-β isoforms in branching morphogenesis and lung development, the studies described above support the concept that tight temporal control of each isoform is critical.

Although temporal regulation of TGF-β isoforms and associated signaling proteins is clearly important for normal lung development, spatial regulation of expression is also crucial. Expression of TGF-β type II receptor (TGFbRII), a receptor that is fundamental to promoting signaling by TGF-β isoforms, is restricted to the airway epithelium in the early embryonic stage (*E11.5*) whereas by the pseudoglandular stage (*E14.5*) expression is found in both epithelial and mesenchymal cell compartments (42). In addition, in the pseudoglandular stage, TGF-β1 gene expression is found within the mesenchyme yet TGF-β2 transcripts are largely absent in the mesenchyme yet present in the distal epithelial, and TGF-β3 transcripts are found in the mesenchyme and mesothelium (43).

Furthermore, evidence of the importance of spatial regulation of TGF-β has been demonstrated in mice with cell-type specific knockouts of proteins crucial to TGF-β activation and signaling. The guanine nucleotide-binding proteins Gαq/11 are crucial for integrin-mediated TGF-β activation in lung epithelial cells (44). Mice lacking Gαq/11 in surfactant protein C (SpC)-positive type 2 alveolar epithelial (AT2) cells have significantly reduced active TGF-β1 and associated Smad2 signaling and develop progressive postnatal alveolar inflammation and lung parenchymal abnormalities, including thickened alveolar walls and increased mean linear intercept (MLI; analysis of airspace size, is inversely proportional to alveolar surface area), together with an obstructive lung function deficit (44). This suggests a critical role for integrin-mediated TGF-β1 activation in maintaining lung homeostasis and normal development postnatally. In addition, mesenchymal cell-specific deletion of Gαq/11 also impacts lung development with mice developing increased MLI, thickened alveolar walls, reduced numbers of secondary crests and abnormal pulmonary vessels by *postnatal day 14*, a phenotype that closely resembles BPD (45). Early evidence suggests a role for TGF-β2 in the development of this phenotype since lung TGF-β2 levels were reduced and knockdown of Gq/11 in human lung fibroblasts reduces expression of TGF-β2 (45). Further research is needed to fully delineate the individual roles of TGF-β isoforms in normal lung development and the pathogenesis of BPD.

In addition to roles for TGF-β isoforms in lung development, research demonstrates that other members of the TGF-β superfamily of proteins are critical during normal lung development and in the pathogenesis of BPD. BMP signaling is active during the later stages of lung development, particularly in the saccular and alveolar developmental stages, and has been heavily implicated in normal branching morphogenesis in the developing lung (30, 46–48). BMP4 in particular has a critical role in normal lung development (32, 49, 50) but lung abnormalities have also been described in mice lacking other functional BMPs including *Bmp5* (51), and in the *Bmp9/10* double knockout mouse (52). Evidence from mouse models of BPD suggests that BMP expression and signaling is reduced (53–55), and recent data demonstrate an inverse correlation between protein levels of bone morphogenetic protein receptor type 2 (BMPR2) and the development of lung structural changes in preterm neonates (54). Furthermore, BMP-9 can protect against impairment of alveolarization in a hyperoxia in vivo model of BPD (56).

BMP signaling is heavily implicated in the development of pulmonary hypertension, which as previously discussed, is associated with BPD pathogenesis. Loss of function mutations in the BMPR2 gene are involved in a large proportion of both familial and idiopathic cases of pulmonary arterial hypertension (57) and genetic mutation of Bmpr2 in rats causes the spontaneous development of pulmonary and cardiac characteristics of pulmonary artery hypertension (58). Functionally active BMPR2 signaling promotes pulmonary endothelial cell survival (59) and targeted delivery of BMPR2 attenuates pulmonary hypertension in rats (60). Crucially, there is crosstalk between TGF-β and BMP signaling pathways (61), meaning that alterations in either TGF-β or BMP levels are likely to dramatically impact both signaling pathways, which could be important in the pathogenesis of BPD.

It is clear from the above-discussed studies that TGF-β isoforms, as well as other members of the TGF-β superfamily, must exist at a tightly controlled equilibrium with under or overexpression leading to impaired lung development and an abnormal lung phenotype, either directly or through interactions with other signaling pathways. Understanding the relationship between antenatal lung development, TGF-β and risk factors in BPD development is therefore key.

## LINK BETWEEN ANTENATAL BPD RISK FACTORS AND ALTERED TGF-β SIGNALING

Although the association between fetal growth restriction or being small for gestational age (birth weight <10th centile) and BPD development is likely multifactorial, they are both recognized antenatal risk factors for the development of BPD (62). Induction of intrauterine growth restriction (IUGR) in rats resulted in impaired alveolar development of the rat pups, which was associated with decreased TGF-β1 expression, downregulation of the TGF-β responsive gene *plasminogen activator inhibitor-1 (PAI-1*) and dysregulation of the composition and remodeling of the ECM components (63). Despite reintroduction of a normal diet at birth and pups displaying catch-up growth, respiratory abnormalities including alveolar simplification and a 30% reduction in MLI persisted. This study supports a separate earlier study in rats showing that IUGR causes decreased TGF-β1 expression (64). Moreover, human placental tissue from pregnancies affected by idiopathic fetal growth restriction has increased expression of transforming growth factor-β-induced factor (TGIF-1) (65), which is a known repressor of TGF-β signaling. Conversely, reports of increased TGF-β expression at *postnatal day 21* in rats with IUGR exist (66) and IUGR in mice causes airway stiffening (67), which is linked with altered TGF-β signaling (68).

Chorioamnionitis is another factor that increases the risk of BPD (8, 69). The relationship between chorioamnionitis, TGF-β, and BPD was explored using intra-amniotic lipopolysaccharide (LPS)-induced chorioamnionitis animal models. Rat pups, whose mothers were injected with LPS on *embryonic day 16.5*, demonstrated pathological features of BPD including fewer terminal air spaces and secondary septa by *postnatal day 7* (70). In sheep, exposure of fetal lambs to intra-amniotic LPS caused an increase in lung TGF-β1 protein and mRNA levels (71, 72) as well as increased Smad2/3 signaling (72–74). In addition, levels of endoglin, a component of the TGF-β receptor complex, are increased in the amniotic fluid of women with chorioamnionitis and overexpression of endoglin in the amniotic fluid of pregnant rats causes decreased alveolarization and vascularization in the rat pups (75).

**Figure 2.**
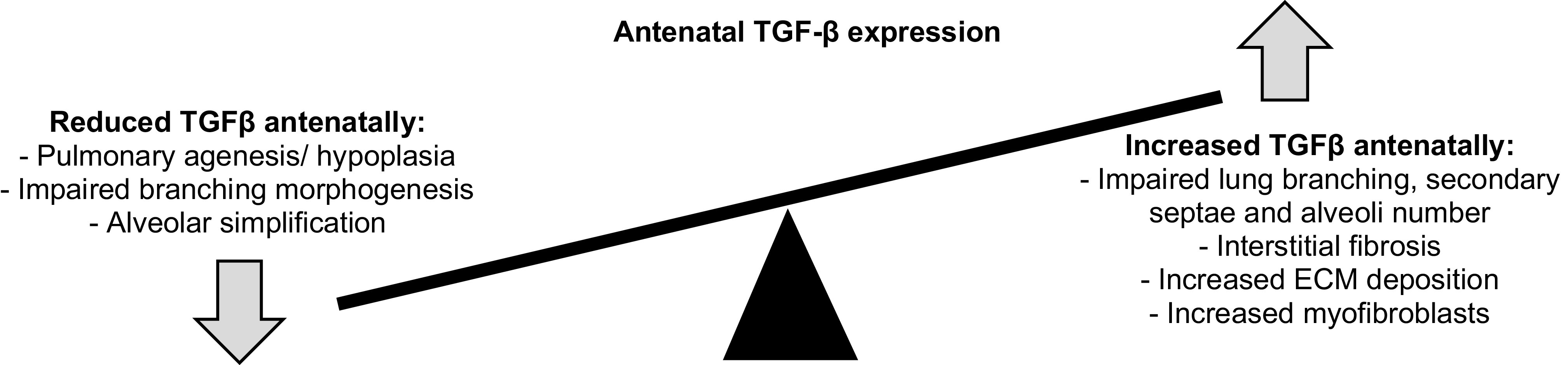
Effect of antenatal under and overexpression of TGF-β on lung development.

As discussed previously, tight control of TGF-β is required to maintain homeostasis and allow correct lung development. The above in vivo animal model studies together with known roles of TGF-β signaling in lung development provide an insight into how disrupted TGF-β signaling antenatally might contribute to aberrant lung development and therefore increased risk of BPD (illustrated in Fig. 2). It is worthy of note that much of the above work has focused on the role of TGF-β1 and much less is known about the relationship between antenatal risk factors and expression and/or activity of TGF-β2 and TGF-β3.

## EFFECT OF POSTNATAL BPD RISK FACTORS ON TGF-β SIGNALING

Mechanical ventilation is an essential treatment strategy in the management of preterm infants; however, there is increasing recognition that their lungs are particularly susceptible to ventilatory-induced lung injury (8), and the need for mechanical ventilation is a well-known risk factor for the development of BPD(76, 77). Early mechanical ventilation in neonatal mice recapitulates the BPD phenotype of abnormal alveolar development with larger, fewer alveoli, increased elastin redistribution throughout the distal airspaces, and increased apoptosis (78–81).

There is now a wealth of evidence supporting a link between mechanical ventilation and altered TGF-β activation in the lungs. Significant correlations between mechanical power of ventilation and levels of TGF-β1 in patients with acute respiratory distress syndrome are evident (82) Neonatal mice exposed to 24 h of mechanical ventilation exhibited a stretch-induced increase in TGF-β activation and a dramatic increase in the TGF-β signaling protein pSmad2 protein in the lungs (78, 80). These effects were also seen in the developed lungs of adult mice who were subjected to volutrauma (expansion-induced injury) outside the period of alveolar lung development (83, 84). Moreover, applying mechanical stretch to ex vivo lung tissue strips activates TGF-β (85). It is proposed therefore that the cyclical stretch of lung tissue involved in mechanical ventilation, a known activator of the TGF-β signaling pathway, is responsible for increased TGF-β signaling and the abnormal lung development and BPD phenotype seen in these animal studies. This is further supported through alveolar SpC-specific deletion of Gαq/11 in mice as described above (84). Here, these mice were not able to generate the increase in TGF-β1 in response to high-pressure ventilation and were protected from ventilator-induced lung injury (84).

Exposure to high amounts of oxygen is another key driver in BPD. Although adequate oxygen is critical for preventing hypoxia, a balance exists to provide adequate oxygen while minimizing oxidative stress (86). Oxygen toxicity is crucial in understanding BPD development and has formed the basis of numerous animal studies. Northway demonstrated severe changes to pulmonary development following exposure of neonatal mice to 100% oxygen with progressive fibrotic lung tissue deposition, bronchitis, bronchiolitis, emphysema and inhibition of lung growth seen (87). Since then, neonatal rodent models have repeatedly demonstrated abnormal lung development in response to hyperoxia with neonatal pups exhibiting alveolar simplification with increased MLI, decreased alveolar number, gas exchange and disordered elastin and collagen deposition (87–95). Over prolonged exposure, animals also developed thickened alveolar septum, excessive α-smooth muscle actin (αSMA) staining, increased myofibroblasts on the septal crests indicative of fibrotic changes (95, 96) and hindered pulmonary microvascular development (88, 94). Recently single cell–sequencing studies have demonstrated that hyperoxia causes dramatic changes in alveolar epithelial cell populations in the lung and alters the transcription profile of genes known to be associated with BPD development, including the protease inhibitor *Slpi* and the immune regulator *Mif* (97, 98). Pathway analysis showed that pathways associated with lung, endothelial, and alveolar development were downregulated in response to hyperoxia (97). Crucially, similar RNA sequencing studies have demonstrated that early life exposure to hyperoxia leads to lasting changes in the cellular composition of the lungs that persist into adulthood (99).

Numerous in vitro and in vivo studies have demonstrated a link between exposure to hyperoxia and TGF-β signaling. Expression of TGF-β1 was increased in vitro in A549 lung cells in a concentration-dependent manner in response to varying oxygen concentrations (40%, 60%, and 95%) (100). Furthermore, multiple in vivo studies have also demonstrated TGF-β overexpression in response to hyperoxia. Mice pups exposed to 85% oxygen from postnatal *days 1*–*20* exhibited increased TGF-β1 expression throughout the alveolar walls and increased pSmad2/pSmad3, suggesting increased TGF-β1 activation. Importantly, administration of intraperitoneal TGF-β neutralizing antibody subsequently dampened phosphorylation of Smad2/Smad3 and resulted in improvements in alveolarization and elastin deposition (91). In separate studies, exposure of mice to 85% oxygen increased mRNA expression of all three TGF-β isoforms, TGFβR1 + 2 and pSmad2/3 (92). TGFβR3, the coreceptor needed primarily for ligand binding of TGF-β2 to the TGFβR2, was reduced. In rats TGF-β1 and ALK5 (aka TGFβR1) mRNA and protein increased alongside a significant reduction in ALK1 and Smad1/5 pathway signaling, suggesting decreased BMP signaling (101).

**Figure 3.**
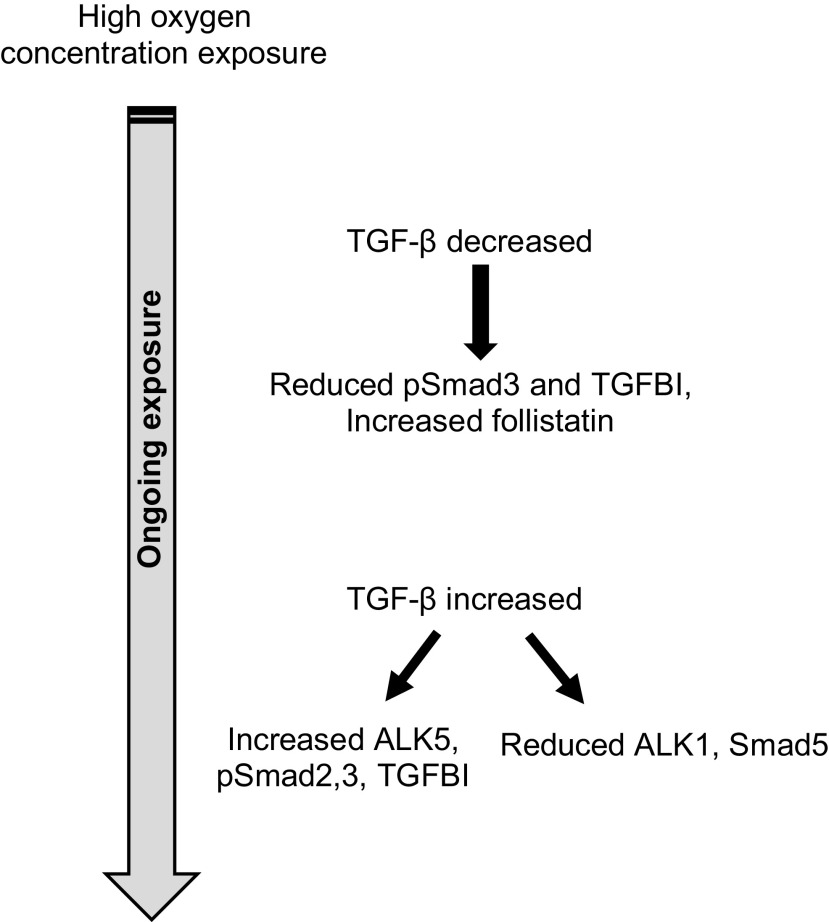
TGF-β expression in response to hyperoxygenation. Initially TGF-β activity decreased in response to hyperoxygenation, however following prolonged exposure, TGF-β activity and downstream signaling increased with increased pSmad2/3.

TGF-β2 may also be affected by hyperoxia. Ahlfeld and colleagues (93, 102) demonstrated varying TGF-β isoform expression and signaling in mice exposed to 85% oxygen (Fig. 3 for overview). Although all TGF-β isoforms were initially reduced, at *day 2* of hyperoxia exposure, TGF-β1 was initially still the predominant isoform; however, by *day 7* during peak alveolar development, TGF-β2 was the predominant isoform. Interestingly here, following continuous oxygen exposure mice subsequently developed TGF-β2, pSmad2, and TGFBI overexpression, as opposed to TGF-β1 in alveolar tissue by *day 14* (104).

Overall, these studies demonstrate that exposure of the postnatal lungs to hyperoxia results in alveolar growth abnormalities in rodents and that there is a growing body of evidence showing a potentially fundamental role for dysregulation of TGF-β isoforms in hyperoxia-induced lung structural changes.

Further in depth understanding of this is key given the established risk of high oxygen exposure and development of BPD in preterm infants.

## IMPACT OF BPD THERAPIES ON TGF-β SIGNALING

There are currently limited treatments in the prevention and treatment of BPD (103), and establishing the best treatment for lung damage in premature infants was identified as a research priority for preterm birth (104). An improved understanding of the mechanism of action of drugs currently in use would help to optimize their use, improve them, and develop more targeted therapies, to ultimately improve the care and treatment of patients with BPD. Current pharmacological therapies available in the prevention and treatment of BPD include caffeine citrate, postnatal steroids, diuretics, azithromycin, and vitamin A (13, 105). Although each has a broad spectrum of physiological and molecular consequences, some may interact with TGF-β signaling.

Caffeine citrate is one of the most widely prescribed drugs in neonatology (106) and reduces the rates of BPD, intraventricular hemorrhage, and neurodevelopmental impairment among preterm infants (107). The Caffeine for Apnea of Prematurity trial for the use of caffeine citrate in preterm infants attributed the increased incidence of BPD among its control group to the extended time this group required positive pressure ventilator support (108). However, there are potentially other effects of caffeine that may explain the decreased BPD incidence with caffeine treatment. Caffeine has been shown to antagonize TGF-β-induced Smad signaling in a concentration-dependent manner in lung epithelial cells and reduced collagen deposition in an ex vivo precision-cut lung slice model of pulmonary fibrosis, suggesting that caffeine inhibits profibrotic effects of TGF-β (109). In animal studies of BPD, mouse lung cells exposed to caffeine demonstrated reduced expression of TGFβR1, TGFβR3, total Smad2, pSmad2, and downstream gene expression (*CTGF* and *PAI*) (92, 110, 111). However, although caffeine normalized Smad2 phosphorylation in hyperoxia-induced BPD mice studies, it was not able to improve the impaired alveolar structure as a result of hyperoxia (92). It is possible that caffeine’s mechanism of action may be multifactorial, working through a combination of reducing apneic events and time requiring mechanical ventilation (thus reducing cyclical stretch-induced TGF-β activation) as well as directly inhibiting the TGF-β activation and signaling itself.

Steroids have a role in the antenatal management of preterm labor (112) and postnatally to reduce the incidence of respiratory disease and BPD in extremely preterm infants (113, 114). Yet the relationship between the use of postnatal systemic corticosteroids, in particular dexamethasone and adverse neurological outcomes, resulted in their use mainly being reserved for infants with severe BPD (115–117). However, a renewed more cautious approach has since begun using early prophylactic steroids to prevent BPD in high-risk infants. Recently a series of multicenter randomized controlled trials (RCTs) have examined the use of early prophylactic low-dose hydrocortisone (118) or inhaled budesonide (119) in high-risk infants to prevent BPD. These both demonstrated a reduction in the incidence of BPD following prophylactic steroid administration (118–121). The use of inhaled budesonide in conjunction with surfactant may offer additional benefits with lower rates of BPD or death compared to those given surfactant alone (42% vs. 66%) (122) with an ongoing RCT (ACTRN12617000322336) further investigating this (123).

Steroids likely exert their effects through multiple biological pathways, including TGF-β signaling. Mice embryonic fibroblasts stimulated with TGF-β1 followed by a glucocorticoid (either dexamethasone, budesonide, fluticasone, or methylprednisolone) exhibited attenuated TGF-β1 activity, demonstrated through reduced activation of the downstream Smad3 binding element, CAGA. Dexamethasone also reduced Smad 2/3 signaling and increased signaling via the TGF-β/Smad 1 axis (124). Dexamethasone in particular may interact with multiple aspects of TGF-β signaling. It was able to interrupt avβ6 integrin expression, a known activator of TGF-β1 which is usually increased in fibrosis in a bleomycin-induced fibrosis animal model (125) and may require TGFβR3 interaction in order to act (124). Using in vitro primary mouse lung fibroblasts, where ablation of the *tgfβr3* gene results in increased TGF-β1-induced gene activation, dexamethasone loses its ability to dampen the effects of TGF-β1 in the knockout cells (124).

However, conflicting results indicate that understanding this interaction is challenging, and that the different isoforms may respond differently to stimulation with steroids. Fehrholz and colleagues assessed the concurrent use of steroids and caffeine in human lung epithelial cells. Here no effect on TGF-β1 mRNA expression was observed in cells treated with either dexamethasone, caffeine or in combination (126). However, there was a small increase in TGF-β2 and TGF-β3 in the presence of dexamethasone with a further rise in TGF-β3 mRNA expression seen when caffeine and dexamethasone were used in combination (126). Overall, dexamethasone appears to influence TGF-β isoform expression, activation, and downstream signaling; however, its exact impact on TGF-β isoform signaling and these translational effects in clinical practice are still to be fully understood.

Retinoic acid and its biologically active form vitamin A are essential for induction of the primordial lung bud in lung development and moderating TGF-β signaling. Disruption of retinoic acid resulted in inhibited lung bud development and increased intracellular pSmad2 and connective tissue growth factor (CTGF) in mice (127, 128). In addition, vitamin A was demonstrated to partially improve alveolar underdevelopment in preterm lambs exposed to mechanical ventilation. In this study, lambs who received daily intramuscular vitamin A developed a heterogeneous lung appearance of both alveolar simplification and more appropriate alveolar formation. They had enhanced blood vessel growth, longer alveolar secondary septae, thinner air space walls, and a greater alveolar number compared to controls. Furthermore, the vitamin A treatment group also had reduced TGF-β activity with reduced pSmad2 on immunostaining and increased vascular endothelial growth factor mRNA (required for vascular development) (129). Vitamin A therefore could be important in promoting correct lung and vascular maturation and reducing the risk of BPD development. In preterm infants, daily intramuscular vitamin A supplementation results in a small reduction in the risk of death and oxygen requirement in BPD (130). However, although it may offer some protective effects against BPD, its intramuscular route of administration and modest clinical benefits likely accounts for this not translating into widespread clinical practice. More recently, inhaled administration has been explored in neonatal rat hyperoxia BPD models. This showed promising results by mitigating the effects of hyperoxia-induced lung damage and enhanced alveolar maturation compared to the intramuscular route (131). This has not been translated into clinical studies.

## EMERGING TREATMENTS IN BPD

Azithromycin is a second-generation macrolide commonly used in the treatment of ureaplasma urealyticum, the most common organism causing chorioamnionitis, a risk factor for BPD development (132). A systematic review and meta-analysis (*n* = 3 studies) showed the use of prophylactic azithromycin at birth led to a significant reduction in the risk of developing BPD [risk ratio 0.86 (95% CI 0.77–0.97)] with a number need to treat of 10 (133). Macrolides have well-described anti-inflammatory properties and may act via a number of mechanisms (134). In bleomycin-induced fibrosis mouse models, mice treated with azithromycin had significantly reduced fibrosis and restrictive lung deficits (135). One mechanism by which azithromycin acts may be through inhibition of TGF-β-induced myofibroblast differentiation (136). In addition, fibroblasts taken from adult patients with pulmonary fibrosis (IPF) exposed to a combination of both TGF-β1 and azithromycin had enhanced antifibrotic and proapoptotic effects compared to TGF-β stimulated IPF fibroblasts (137). Although we found no published studies on azithroymcin and TGF-β signaling in relation to BPD the above studies suggest there is merit in further research in this area. In the United Kingdom, a large multicenter randomized controlled trial has completed recruitment (ISRCTN11650227) assessing the effectiveness of a 10-day course of prophylactic azithromycin from birth in infants less than 30 wk, with the primary outcomes of diagnosis of BPD and mortality at 36 wk postmenstrual age (138).

Stem cells are a potentially exciting therapeutic strategy in regenerative medicine. Studies have moved over the past 10 year from initial proof of concept studies toward recruitment for RCTs (NCT03645525, NCT03392467) (139–142). In humans, a Phase I trial delivered intratracheal human umbilical cord blood-derived mesenchymal stem cells (MSCs) to preterm infants at high risk of developing BPD. Although this was a feasibility study with a small sample size, no infant in the treatment group was discharged home with supplemental oxygen (compared with 22% in the control group). Furthermore, a reduction in proinflammatory cytokines including TGF-β was seen in tracheal aspirates of infants in the treatment group by *day 7* (143, 144). A Phase II trial also using intratracheal administration of MSCs showed similar promising results, with a reduction of severe BPD in infants born at 23–24 wk gestation (19% BPD in the intervention group vs. 53% BPD in the control group) (145). Animal studies have shown improvements in the pulmonary architecture of animals following MSC administration. MSC administration reduced oxygen-induced lung damage, inflammation, and fibrosis (146–148) whereas intraperitoneal administration of human amnion epithelial cells reduced alveolar simplification and improved body weight in mice (147). Stem cells could also dampen TGF-β1 expression and downstream signaling in BPD animal studies (146, 148).

## CONCLUSIONS

TGF-β is a complex and important cell signaling pathway implicated in a number of respiratory and fibrotic disease pathways and plays a key role in BPD development. The correct balance of TGF-β isoform expression, activation, and downstream signaling is essential for normal lung development and can be influenced by multiple risk factors implicated in BPD development. Current treatments already in use in neonatology may exert their mechanisms of action, at least in part, through modulating TGF-β signaling. However, most of the research currently investigating this is limited to in vitro and rodent animal models with very few studies in larger animals or translated into clinical practice. More research and understanding of this important cell signaling pathway and its interaction with other related pathways could be further explored and aid in the development of more targeted treatment strategies for use in the management of BPD.

## GRANTS

This work was supported in part by Medical Research Foundation Grant MRF-091-0004-RG-TATLE to A.L.T. and by a National Institute for Health and Care Research (NIHR) Academic Clinical Fellowship to R.J.C.

## DISCLOSURES

A.R.S has research grants (paid to the University of Nottingham) from Vertex Pharmaceuticals and payment for an advisory board (paid to the University of Nottingham) from Viatris Pharmaceuticals, all outside the current work. A.R.S. has patents issued (Camara M, Williams P, Barrett D, Halliday N, Knox A, Smyth A, Fogarty A, Barr H, Forrester D. Alkyl quinolones as biomarkers of Pseudomonas aeruginosa infection and uses thereof. US2016131648-A1; https://pubchem.ncbi.nlm.nih.gov/patent/US-2016131648-A1). Outside the current work, A.R.S reports participation on a Data Safety Monitoring Board for the North American Cystic Fibrosis Foundation Therapeutic Development Network. R.J.C. was previously supported by a National Institute for Health Research (NIHR) Academic Clinical Fellowship. G.J. reports personal fees and other from Biogen, personal fees from Galapagos, other from Galecto, personal fees and other from GlaxoSmithKline, personal fees from Heptares, personal fees and other from MedImmune, personal fees from Boehringer Ingelheim, personal fees from Pliant, personal fees from Roche/InterMune, personal fees from PharmAkea, personal fees from Bristol Myers Squibb, personal fees from Chiesi, personal fees from Roche/Promedior, other from RedX, other from NuMedii, other from Nordic Biosciences, personal fees from Veracyte, outside the submitted work. G.J. is supported by a National Institute of Health Research Professorship and is a trustee for Action for Pulmonary Fibrosis. A.L.T. reports consulting for biotech.

## AUTHOR CONTRIBUTIONS

R.J.C. and C.P. prepared figures; R.J.C., C.P., and A.L.T. drafted manuscript; R.J.C., C.P., A.R.S., D.S., J.B., G.J., and A.L.T. edited and revised manuscript; R.J.C., C.P., A.R.S., D.S., J.B., G.J., and A.L.T. approved final version of manuscript.
